# The head and neck cancer (HN-5) cell line properties extraction by AFM

**DOI:** 10.1186/s13036-020-00233-6

**Published:** 2020-03-18

**Authors:** M. H. Korayem, K. Heidary, Z. Rastegar

**Affiliations:** 1 Robotic Research Laboratory, Center of Excellence in Experimental Solid Mechanics and Dynamics, School of Mechanical Engineering, Iran University of Science and Technology, Narmak, Tehran, 16846 Iran; 2 Department of Computer Engineering, Islamic Azad University, Sciences & Research Branch, Hesarak, Tehran, 1477893855 Iran; 3 School of Mechanical Engineering, Iran University of Science and Technology, Narmak, Tehran, 16846 Iran

**Keywords:** Characterization, AFM, Biological particles, Modulus of elasticity, Viscoelastic, Mechanical properties, HN-5

## Abstract

This work incorporates experimental methods based on Atomic Force Microscopy (AFM) in order to extract the physical and mechanical characteristics of the head and neck cancer (HN-5) cell line such as cell topography, modulus of elasticity and viscoelastic properties. The initial parameters to determine the mechanical properties are obtained by extracting information from cantilever’s force-displacement curve and vertical and horizontal displacement. Next, the changes in elasticity modulus at different points in the cell are attained using the experimental results, followed by studying the differences of these properties at various spots of the cell. Furthermore, cellular folding factor is calculated as a significant property in diagnosing the extent of cancer progression. Moreover, parameters such as adhesion and intermolecular forces are measured which are involved in the first phase of manipulation and during the application of the cantilever force to the particle. Finally, after calculating the indentation depth and contact radius using contact theories, critical manipulation time and force are obtained. Through modeling the cell, the creep function, the spring constant and the damping coefficient corresponding to the cell, are also extracted.

## Introduction

Mechanical models are useful tools to identify cancer cells and viruses accurately and to predict the biological particles’ behavior should be explored. These models help to understand the topography of biological cells, as well as the physical and mechanical characteristics such as: elastic modulus, adhesion and viscoelastic properties of the cell.

Vinckier and Semenza calculated the elasticity module of biological cells and soft matters using AFM and extracted a method to measure elasticity based on the Hertz Model [1]. Prabhune et al. studied the mechanical properties of normal and malignant thyroid cells using AFM and showed that malignant thyroid cells are 3 to 5 times softer compared to primary normal thyroid cells. These results highlighted the importance of cultivation period influencing various kinds of cells’ mechanics [2]. Sirghi et al. showed that an appropriate indentation model can provide information on the cell cytoskeleton elasticity and adhesion of the cell membrane to the surface of the AFM probes [3]. In another study, they reported the effect of indenter-cell adhesion force on force-displacement curves and how an appropriate indentation model is able to provide useful information on the cell elasticity and the work of adhesion between the cell membrane and AFM probes [4]. Li et al. showed that the mechanical properties of individual living cells are closely related to the health of the human organization. The elasticity of benign (MCF-10A) and cancerous (MCF-7) human breast epithelial cells was characterized by AFM indentation using a micro-sized spherical probe and it was shown that malignant (MCF-7) breast cells have an apparent Young’s modulus significantly lower (1.4–1.8 times) than that of their non-malignant (MCF-10A) counterparts at physiological temperature (37 °C) where the Young’s modulus increased with loading rate [5]. Zhou Z. et al. used AFM to extract the elastic modulus of tongue squamous carcinoma cells (TSCC) with different metastatic potentials. It was found that TSCC cells with higher metastatic potential showed lower elastic modulus values compared to TSCC cells with lower metastatic potential. In addition, they obtained elastic modulus values of 7.55 ± 1.71 kPa for the first tissue sample used in the study [6]. Utilizing a tip-less cantilever, Puech et al. measured adhesion forces of zebrafish single cells to substrates and extracted maximum separation force and work of adhesion from the force-displacement curves [7].

Gascoyne and Shim indicated that the cell plasma membrane of most cell types is not smooth but contains different surface features including microvilli, folds, and ruffles that cause mammalian cells to have larger membrane surface areas than ideally smooth spheres of similar volume. In order to quantify the surface area difference, they introduced the concept of a membrane folding factor, ϕ, the ratio of actual cell membrane area to the area of a perfectly smooth cell of similar volume [8]. Using AFM, Korayem et al. studied the topography of MCF10 cells in order to extract the folding factor of cells experimentally. By applying this factor in the Hertz, Derjaguin–Muller–Toporov (DMT), and Johnson-Kendall-Roberts (JKR) contact models in the elastic and viscoelastic states, these models have been modified and indicated that the simulation results converge to the experimental results by considering the folding in the calculations [9]. In another study, they used elastic and viscoelastic JKR theories in modeling and simulation of the 3D manipulation for three modes of tip–particle sliding, particle–substrate sliding and particle–substrate rolling. Results showed that the critical force and time in sliding and rolling modes for two elastic and viscoelastic states are close but lower in the viscoelastic state [10].

Using AFM E. A-Hassan et al. indented soft samples for providing information about the local viscoelasticity [11]. By investigating the local viscoelastic characteristics of fibroblast cells, Cartagena and Raman locally measured stiffness and viscoelastic properties and attained the difference between the spring force and viscosity of the material [12]. In a quantitative analysis of the cell-surface roughness and viscoelasticity for breast cancer cells discrimination using AFM, Wang et al. characterized and compared the surface nanostructure and viscoelasticity of different breast cell lines. Their results showed that MCF-7 breast cancer cells exhibit more disorganized filamentous cytoskeleton structure with increased membrane roughness compared to benign breast cells MCF-10A (*P* < 0.05). The viscoelastic properties were significantly different between the two cell lines. MCF-7 displays reduced elasticity and viscosity, indicating that breast cancer cells are softer than benign counterpart [13]. Korayem et al. developed and modeled a number of contact theories for biological nanoparticles shaped as cylinders and circular crowned rollers for application in the manipulation of different biological micro/nanoparticles using AFM. They adapted the contact theory in accordance with the geometry of the particles to the extracted force-displacement curve via AFM and calculated the critical time and force in the manipulation using the indentation depth and contact radius [14].

The current study is the first step toward characterizing cancerous cells mechanical properties from which the Head and Neck cancerous (HN-5) Cells in a heterogeneous population can be identified. In this study, in addition to cell geometry and topography, most of the mechanical properties of the HN-5 cells line such as the Young’s modulus measured from experimental results are derived. Cell adhesion force and its variation at points with different thicknesses, viscoelastic properties and the changes with the depth of indentation, intermolecular force of the sample, the energy dissipation rate, the coefficient of stiffness and the amount of the cell deformation according to the force-depth curve are calculated. The methods of calculating folding factor of the cell are further improved to obtain a more accurate factor. Furthermore, the creep function is modeled to estimate the viscoelastic behavior of the biological cell more accurately. Finally, the first phase of 3D manipulation for HN-5 cells is simulated.

## Experimental methods

### Preparing the equipment and cell for the experiment

**Fig. 1:**
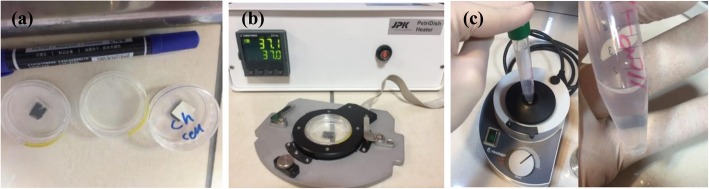
**a** Prepared Petri dishes, **b** Petri-dish heater device, **c** Preventing the deposition of cells in Falcon

The AFM device used in this work is the Nanowizard II atomic microscope manufactured by JPK (JPK Instruments Inc., Germany) with the scan range from 1 nm to 100 μm which is ideal for soft material investigations as well as interaction studies. It supports contact and tapping modes and can be used in vacuum, gas (air) and liquid environments. The probes are made of soft silicon nitride cantilevers with reflective gold back side coating and silicon nitride tips with the height and radius of 2.5–8.0 μm and 10 nm, respectively. Moreover, the spring constants of the cantilever varies in the 0.025–0.14 N/m range and its nominal stiffness is 0.07 N/m. The biological particles used in this paper are the head and neck cancer (HN-5) cell line and analyzed under fixed conditions. The HN-5 cell is derived from tongue squamous cell carcinoma and its preferred name is “Tongue Squamous Cell Carcinoma”. After their revival, the cells are placed in 3 petri-dishes and installed for AFM experiment (Fig. 1a). The petri-dishes are prepared beforehand such that each is coated with a different material - MgCl_2_, mica, and silicon - to paste the buffer and cells to the surface.

It is crucial that the cells are in their physiological conditions, by keeping them at 37 °C using a petri-dish heater shown in Fig. 1(b). To prevent the cells from agglomeration, the falcon containing the cells is shaken using a vortex mixer, to allow the homogenous distribution of the cells in the buffer medium, as shown in Fig. 1(c). After installing the equipment and starting the experiment, it is necessary to observe the images of the cells to ensure that they are not agglomerated and their relative position is obtained. To perform the test, it is necessary to mount and align spherical probes on the AFM head and calibrate the cantilever. There was a set of cells in each Petri dish and the indentation was performed in at least 10 cells for each of the 3 Petri-dishes and repeated for at least 10 different points of the cells for the two contact modes.

The extend/retract speed was set to 5 μm/s using closed loop and the force-distance curves were extracted directly above the nucleus of the cells. In addition, the data is recorded for the both contact and tapping modes. The test generally includes two steps: first, cell positioning and second, the acquisition of indentation curves. In the processing phase, the initial step is to remove any offset or tilt from the curve and find the contact point; therefore, the options ‘Subtract Baseline’ and ‘Find the Contact Point’ are to be selected. The following step is to correct the height for cantilever bending, a feature that measures the indentation depth by taking the difference between the piezo movement and the cantilever vertical deflection in units of length. Finally, the curves are ready to be fitted with the Hertz model to calculate the Young’s modulus. The DMT model, which considers adhesion forces in the contact area, can also be used for the tips with low curvature radius and high stiffness.

## Modeling of biological particles

**Fig. 2:**
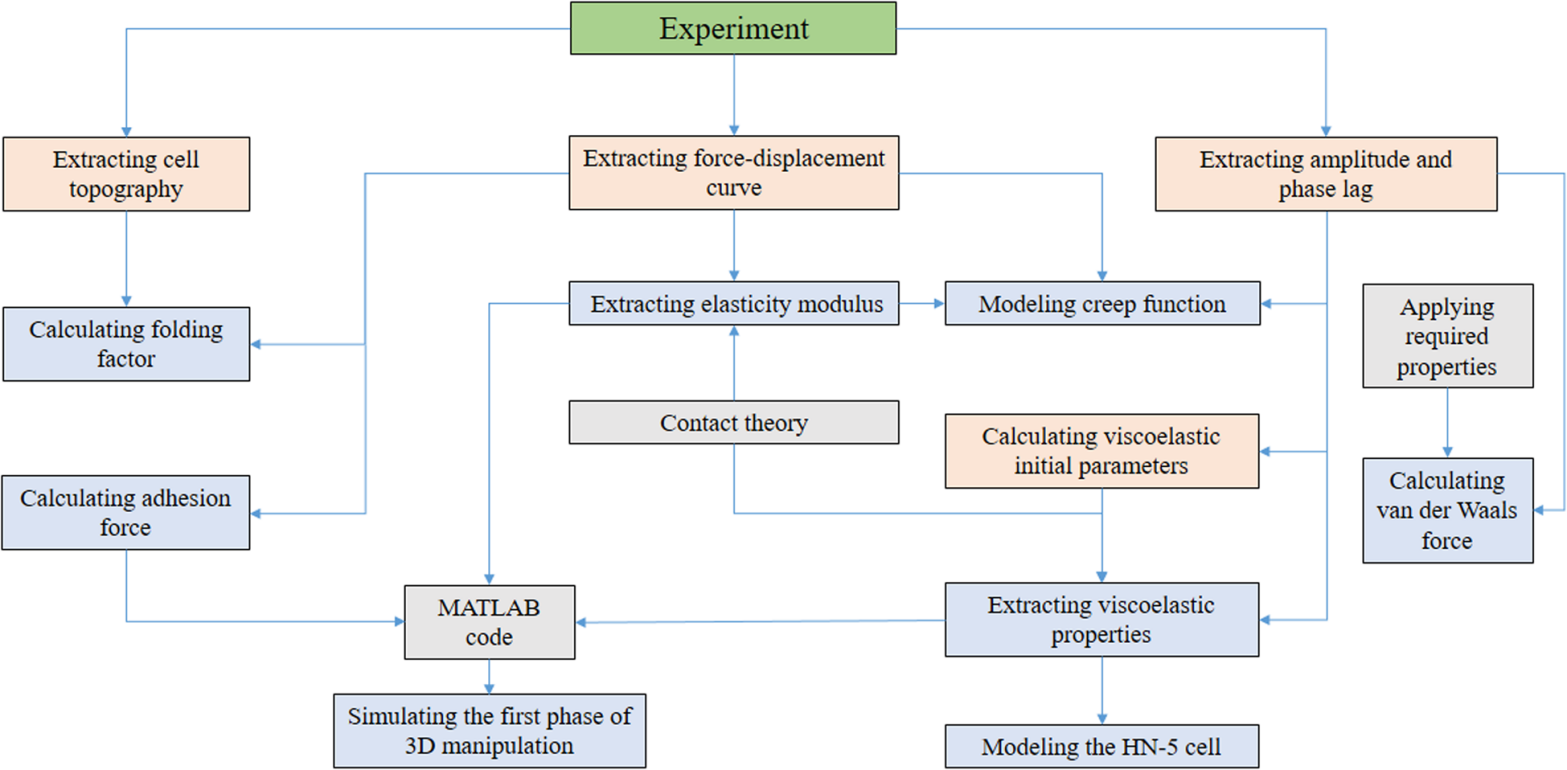
The flowchart of the study

The cell’s mechanics includes a variety of features including cell shape and movement, adhesion force, deformation ability and properties such as elastic modulus, viscoelastic coefficients and creep function. One of the fundamental goals of studying cell mechanics is to explain the mechanical properties of cells, cell structures and the mechanical effects of cells on one another and their surroundings. The following is a brief description of the features that can be used to describe the factors involved in cell mechanics. Furthermore, the procedure of conducting the experiment is presented in Fig. 2.

### Modulus of elasticity

**Fig. 3:**
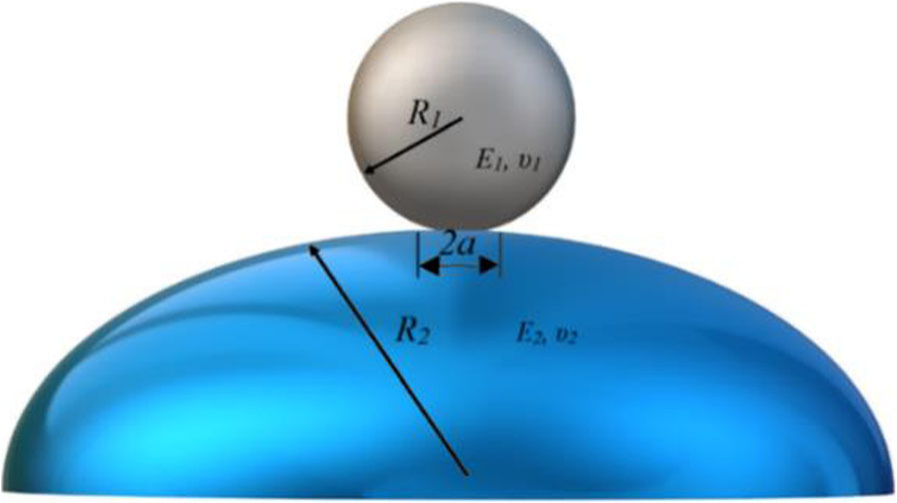
Schematic representation of the contact between an AFM spherical tip and a cell

By inserting the force curve data and indentation depth extent into Hertz elastic theory, the elasticity modulus of each cell is obtained at each point. For contact between two spheres of radii *R*_*1*_ and *R*_*2*_ (Fig. 3), the effective radius *R* is defined as *R* ≡ *R*_1_*R*_2_/(*R*_1_ + *R*_2_). Also, if the elastic modulus of the two particles are respectively *E*_*1*_ and *E*_*2*_, the effective elastic modulus is defined as ${E}^{\ast}\equiv {\left(\left(1-{\nu}_1^2/{E}_1\right)+\left(1-{\nu}_2^2/{E}_2\right)\right)}^{-1}$, where υ is the Poisson’s coefficient [15].

### Folding factor for HN-5 cell line

In their natural state, the cells have a folded and uneven surface and the roughness on the surface of the cells depends on the amount of damage [8, 9]. It is possible to examine cell surface and measure shape and volume by extracting the topography of the sample. The folding factor of the cell is obtained by taking the area of the damaged cell and dividing it by the area of the smooth cell that has the same volume as the damaged cell. Since the cells do not have a smooth surface in their real shape, the value of this factor must be greater than 1.

### Viscoelastic properties

The Kelvin–Voigt is one of the most important rheological models and is used for the modeling of viscoelastic and creep functions. The model is derived from a viscous damper and an elastic spring connected in parallel which makes the strains in each component to be equal. Therefore, it is possible to make an equilibrium for forces or pressures on connections and the total stress is the sum of the stress in each component. By Eqs. 1 and 2, ${k}_{sample}^{dynamic}$ and ${c}_{sample}^{dynamic}$ that represent the parameters of a Kelvin-Voigt element, can be attained [16].

1 $$ {k}_{sample}^{dynamic}=\left(\frac{k_{cant}{A}_{1 far}}{Q_{far}{A}_1}\cos \left({\phi}_1\right)-\frac{k_{cant}{A}_{1 far}}{Q_{far}{A}_{1 near}}\cos \left({\phi}_{1 near}\right)\right)\times \sqrt{1-\frac{1}{4{Q}_{far}^2}} $$

2 $$ {c}_{sample}^{dynamic}=\left(\frac{k_{cant}{A}_{1 far}}{Q_{far}{A}_1}\sin \left({\phi}_1\right)-\frac{k_{cant}{A}_{1 far}}{Q_{far}{A}_{1 near}}\sin \left({\phi}_{1 near}\right)\right)\times \sqrt{1-\frac{1}{4{Q}_{far}^2}} $$

In these equations, *A*_1far_ is the oscillation amplitude, *ϕ*_1far_ is the phase lag far from the sample surface, as the oscillation amplitude and the phase lag near the surface are *A*_1near_ and *ϕ*_1near_. *ω*_far_ is the cantilever frequency (rad/s), *Q*_far_ is the quality factor far from the sample surface and *k*_cant_ is the calibrated cantilever spring constant. Besides, micro-cantilever records three channels of information: mean deflection (*A*_0_), first harmonic amplitude, and phase lag (*A*_1_ and *ϕ*_1_).

### Van der Waals forces

In order to simulate contact mechanics in biological environments, intermolecular forces should also be considered. One of these forces is the van der Waals force. Before using the required equations to calculate the van der Waals force, the Hamaker constant should be defined. The Hamaker constant (*A*) for the two environments 1 and 2, which are located in the environment 3, is expressed in Eq. (3), where k is the Boltzmann constant, *T* is the absolute temperature, *ε*_1_ is the dielectric constant of the tip, *ε*_2_ is the dielectric constant of the sample and *ε*_3_ is the dielectric constant of the medium [17].

3 $$ A\cong \frac{3}{4}{k}_BT\frac{\left({\varepsilon}_1-{\varepsilon}_3\right)\left({\varepsilon}_2-{\varepsilon}_3\right)}{\left({\varepsilon}_1+{\varepsilon}_3\right)\left({\varepsilon}_2+{\varepsilon}_3\right)} $$

While the tip approaches the sample, van der Waals force can be obtained. Furthermore, the most accurate value for the desired distance in the van der Waals equation is derived from the force-displacement diagram in the extend mode. Moreover, to calculate the van der Waals force, we use the Eq. (4), rewritten for the force between AFM tip and sample force, in which *a* is the radius of the tip, *h* is the distance between the tip and the substrate, and *A* is the Hamaker constant.

4 $$ {F}_{vdw}(h)=\frac{A}{6}\left[-\frac{a}{h^2}-\frac{2a}{h{\left(h+2a\right)}^2}+\frac{2a}{h\left(h+2a\right)}\right] $$

### Creep function

The creep function for a cell simulated by a simple viscoelastic model such as Kelvin–Voigt model, with a spring and a damper in parallel, will be in the form of Eq. (5) [18].

5 $$ {C}_{sample}(t)=\left(\raisebox{1ex}{$1$}\!\left/ \!\raisebox{-1ex}{${E}_{sample}$}\right.\right)\left(1-{e}^{\raisebox{1ex}{$-t$}\!\left/ \!\raisebox{-1ex}{${\tau}_{sample}$}\right.}\right) $$

In a viscoelastic material, the creep compliance curve in terms of time shows that in a constant stress, compliance (or strain) increases over time. The study area in the creep test is related to the part of the delayed elastic strain; meaning that if the stress is eliminated, the sample will return to its original state over time *t*. To obtain the creep function, it is necessary to extract the time component from the experiments.

## Experimental results and discussions

### Horizontal and vertical displacement profiles

**Fig. 4:**
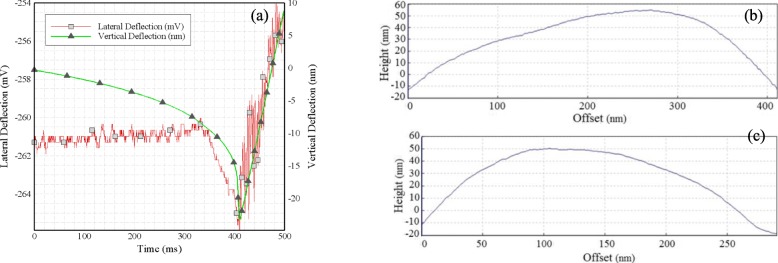
**a** Vertical profile and horizontal displacement in terms of time, **b** Height profiles on the large diameter of the cell, **c** Height profiles on the small diameter of the cell

The horizontal and vertical displacement profiles are the two main outputs of the experiment which can be recorded in real time (Fig. 4(a)). Another important extracted component is the height profile of the cell in different lengths and widths. For this cell sample, the maximum recorded width is 291 nm, the maximum length is 432 nm, and the maximum height is 53 nm.

**Fig. 5:**
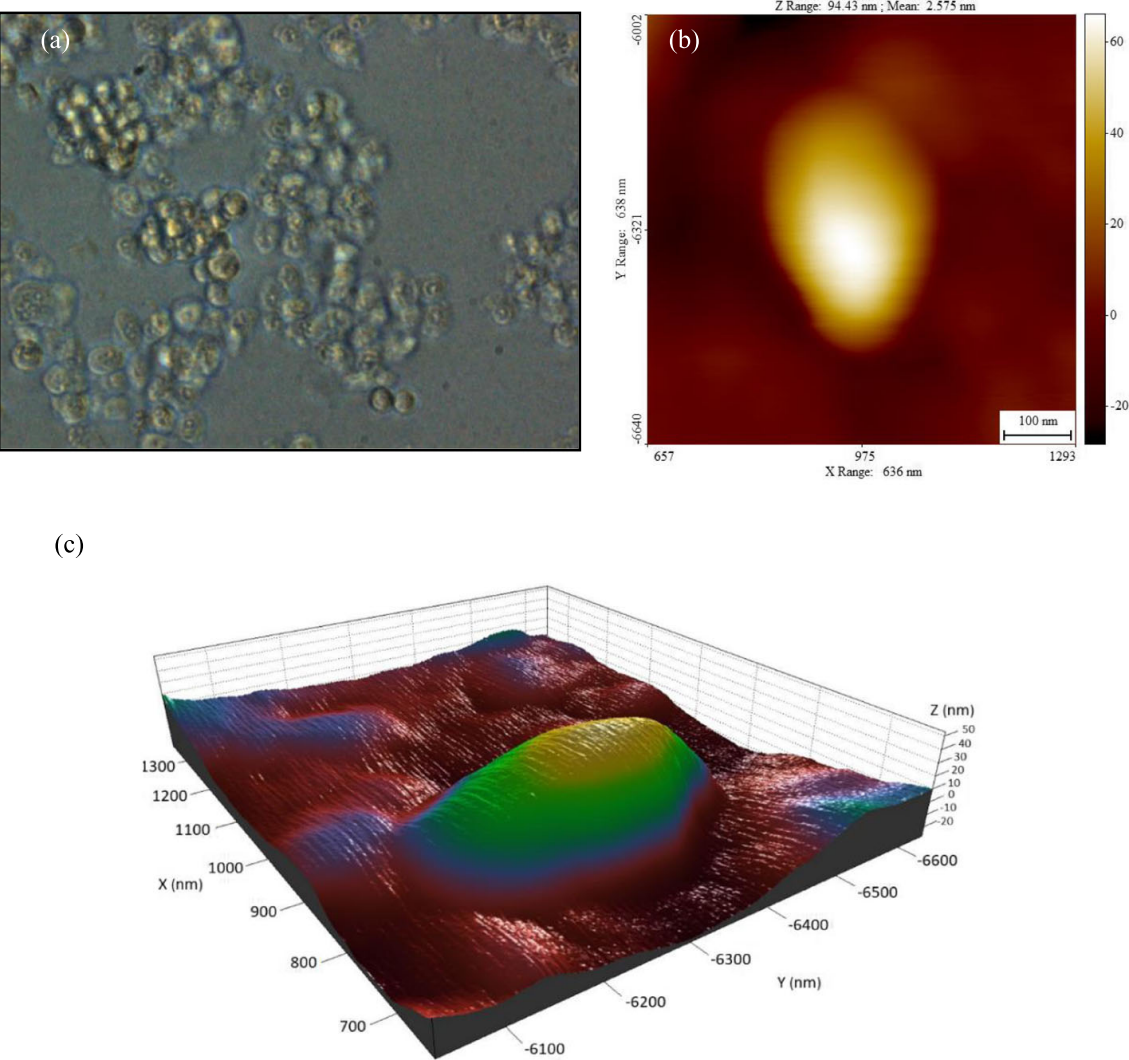
**a** The first image taken from the initial scan of the cell sample, **b** 2D image of the cell in tapping mode and **c** 3D image of the HN-5 cell using the SPIP software

In Fig. 4(b), (c) the graphs acquired from the height profile for the large and small diameter of the cell are presented. In addition to the obtained values, Fig. 5(a) is an image taken from a primary scan of the cell sample and Fig. 5 (b) shows 2D image of the cell in tapping mode for that the maximum recorded height of the cell, in this case, is 52 nm and the large and small diameters of the cell are 234.7 and 352.9 nm. Moreover, the 3D image of HN-5 cells is extracted using the tools available in the Scanning Probe Image Processor (SPIP) software as shown in Fig. 5 (c).

### Measurement of elasticity modulus by experimental methods

**Fig. 6:**
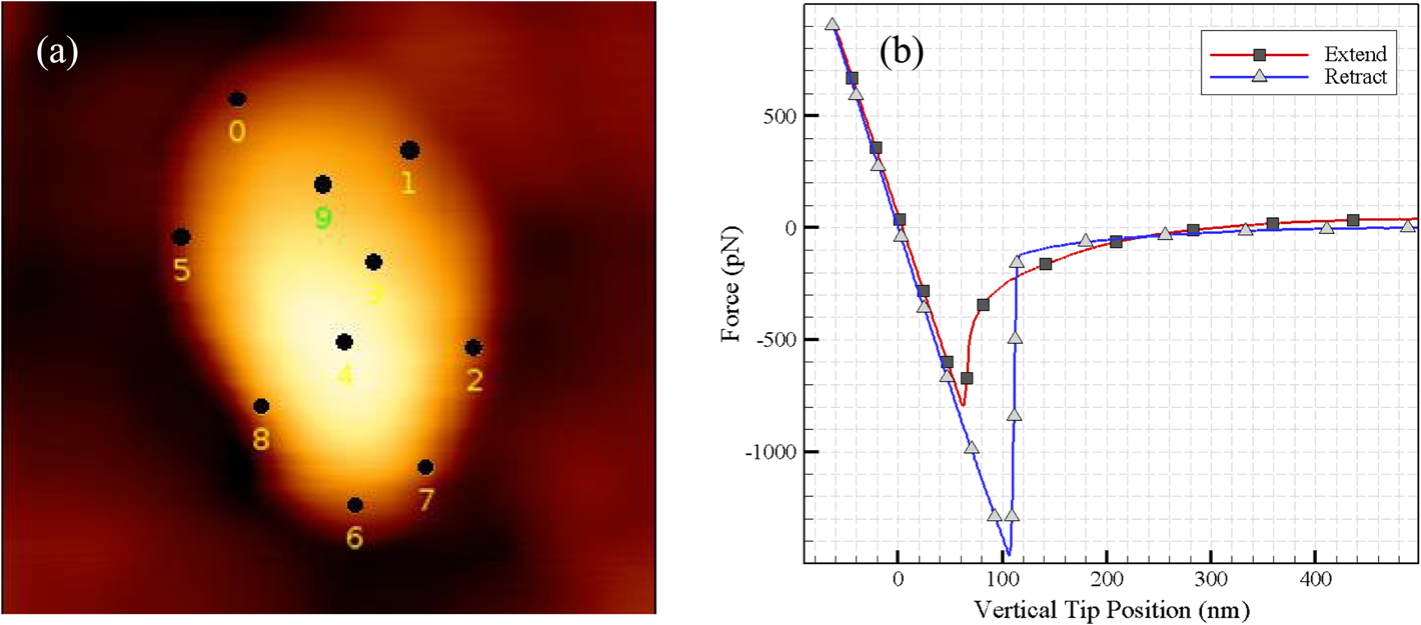
**a** Selected points to extract Young’s modulus and **b** The measured force of the cantilever’s extend and retract

**Fig. 7:**
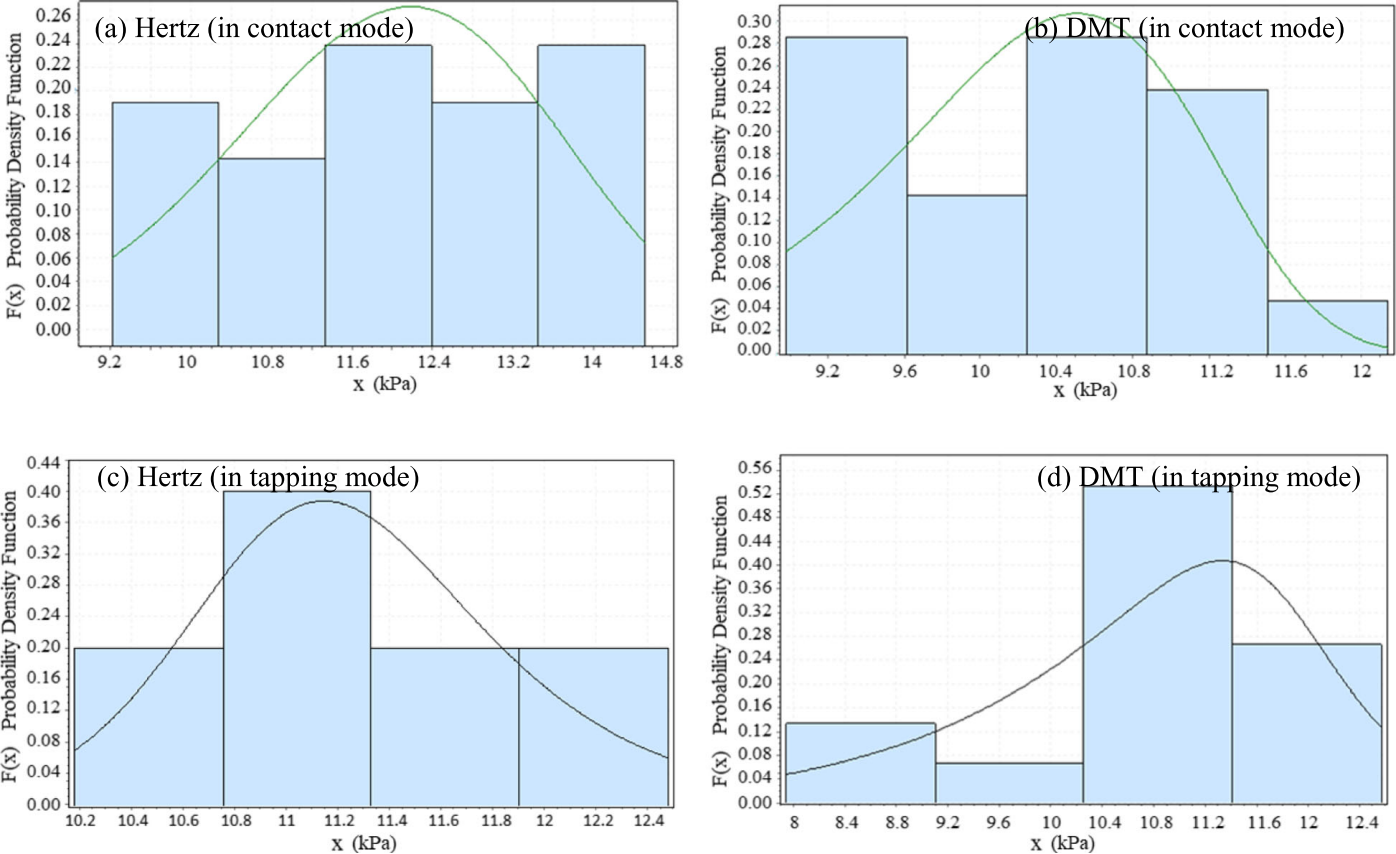
The normal distribution and histogram of the calculated Young’s modulus in contact mode for **a** Hertz, **b** DMT contact models and in tapping mode for **c** Hertz and **d** DMT contact models

To calculate the Young’s modulus, the indentation was repeated for 10 points and the modulus parameters were first calculated by the Hertz contact model, then by DMT contact model for each repetition. Figure 6 (a) shows selected points to extract Young’s modulus and Fig. 6 (b) presents an example of the cantilever’s extension and retraction on the tested cell in selected points. The process of characterization is repeated for tapping mode. Next, the normal distribution and the histogram of the results are calculated and plotted for Hertz and DMT contact models as presented in Fig. 7(a-d). As shown in the tapping mode and Hertz contact model, the Young modulus has more repeatable results, although the results are more dispersed when using DMT contact model.

In this experiment, ellipsoid geometry is considered for the cell and Poisson’s ratio is set to 0.5 as used generally for soft biological particles. A spherical probe with a Poisson’s ratio 0.25 is mounted on the cantilever since for the biological samples, it is recommended to use spherical probes since the force is applied to a wider sample area. The average Young modulus for this sample for contact and tapping modes in the air environment is presented in Table 1. These quantities are obtained by fitting the indentation results with Hertz and DMT contact models. In the test, the indentation was performed repeatedly in at least 10 different points and for the two contact modes. Next, using the Hertz and DMT’s elastic theory, the Young modulus of the cell was calculated at its various points. Points 3, 4, and 9 in Fig. 6 (a) have the maximum recorded height and the side points of the cell have the minimum thickness.

**Table 1:** Extracted Young’s modulus for Hertz and DMT contact models in contact and tapping mode

Young modulus (kPa)	Contact/ Hertz	Contact/DMT	Tapping/Hertz	Tapping/ DMT
Min	9.21	8.98	10.18	7.94
Max	14.52	12.14	12.48	12.57
Mean	11.95	10.29	11.28	10.72

The results show that the elasticity modulus is higher in spots with less altitude and lower in spots with higher altitude. Additionally, as shown in Table 1, Young’s modulus extracted from the DMT contact model is less than the Hertz model and this pattern is similar for different points of the cell in contact and tapping modes of the AFM. Since thicker regions located at the center of cell contain more liquid and should be softer compared to the edges, and considering the range of Young’s modulus in the relevant articles, these results are as expected. Moreover, substrate effects can be considered as another factor in increasing the stiffness along the cell periphery. The reproducibility of the results in this experiment, in addition to them being in similar ranges as in others studies, can be attributed to their validity [19–22]. Zhou Z. et al. obtained the elastic modulus of 7.55 ± 1.71 kPa for the first tongue squamous carcinoma cells (TSCC) sample they used in the study. These values are consistent with our results, since the range of the modulus obtained in our study is 7.94–14.52 kPa [6].

### Force and work of adhesion

**Fig. 8:**
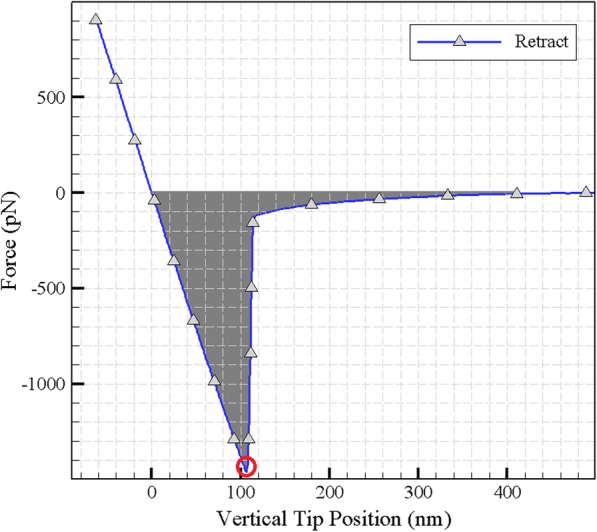
Calculating the force and work of adhesion according to the parameters measured in the retract period

It was previously described that the retracting part of the force-distance curve reveals the maximum adhesion force as shown in Fig. 8. The amount of adhesion work which can be obtained by calculating the integration of the indentation force over the displacement of the cantilever in the retract period is calculated to be about 98,706 × 10^− 21^ J.

**Fig. 9:**
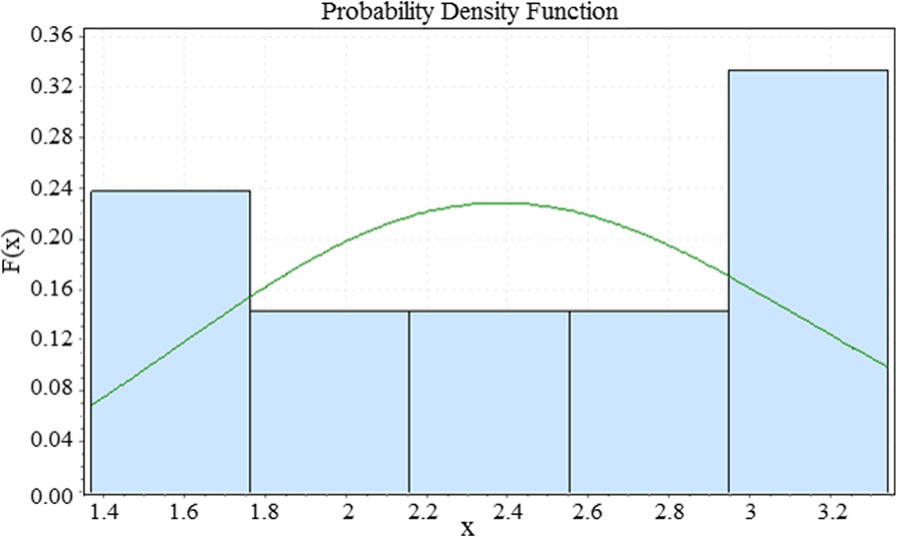
The histogram of the extracted values for the adhesion force in contact mode

Figure 9 shows the histogram of the extracted values for the adhesion force.

The purpose of this section is to determine the amount of adhesion force variation in different spots of the cell in order to make a comparison and calculate the difference of this cell’s properties from point to point. It should be noted that the measurements were taken in air and the points measured for adhesion are those used in Young’s modulus. This helps to smooth out the test and give more accuracy to the assessments. The average adhesion value for air medium and cantilever’s contact mode is 2.47 nN. The adhesion force-distance during the retract time retains an analogous trend for all measurements taken. Similar to the modulus of elasticity, the maximum adhesion was observed on the sides of the cell and the minimum value was observed in the middle. These results indicate that softer sections of the cell are more adhesive (Table 2).

**Table 2:** The adhesion force extracted for HN-5 cells

The adhesion force	nN
Min	1.37
Max	3.34
Mean	2.47

#### Folding factor (first method)

**Fig. 10:**
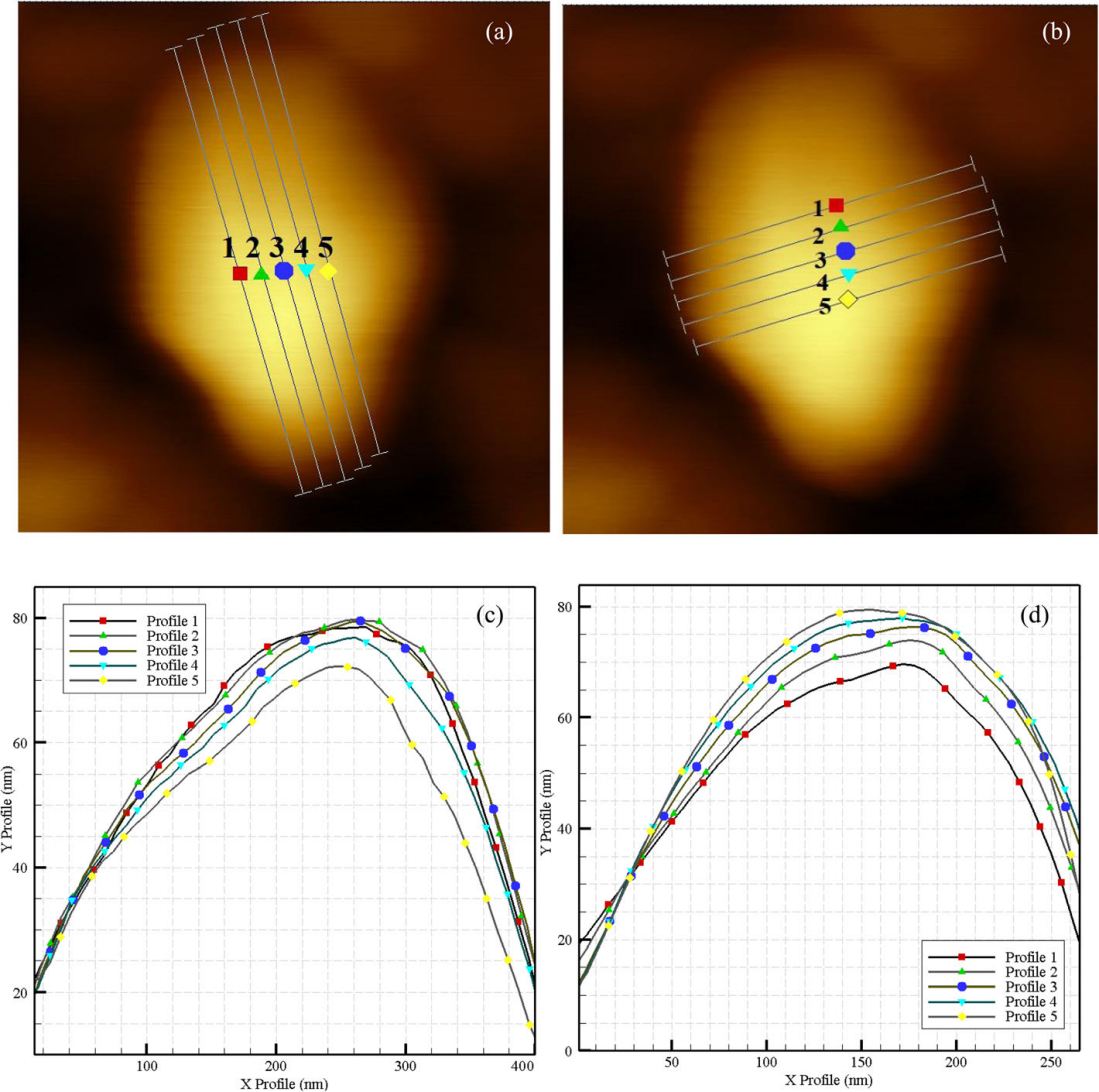
**a** 5 approximation lines for large diameter and **b** Small diameter of the cell (scale bar), **c** Height profiles for each of the lines on the large and **d** Small diameters of the cell

First, the folding factor of the cell is extracted in contact mode and air environment. In order to make an appropriate approximation, as shown in Fig. 10 (a, b), roughness and height parameters are obtained for 5 lines of large diameter and 5 lines of small diameter. In Fig. 10 (c, d), the height profiles are shown for each of the lines on the large and small diameter.

Moreover, the measured values for each of the graphs are given in Table 3, in which the exact values of the length of the lines, their height, and the area under the curves are presented.

**Table 3:** The length and height of the lines and the area under the curves for the (a) Large diameter and (b) Small diameters of the cell in nm. (c) The corresponding folding factor of the lines on the diameters and average folding factor for HN-5 cells

	1	2	3	4	5	Mean
(a) Large diameter						
Maximum	44.43	44.187	43.217	42.124	39.811	42.7538
Average	26.964	25.157	24.441	23.458	22.578	24.5196
Median	31.337	29.519	28.08	27.181	24.747	28.1728
Ra	13.299	14.037	13.467	12.77	11.329	12.9804
Rms (Rq)	15.43	16.439	15.873	14.987	13.301	15.206
length	408.59	426.04	427.25	421.28	401.27	416.886
Developed length	442.13	460.38	461.19	453.87	430.73	449.66
Positive Area under curve	11,196.1	10,730.5	11,078.6	10,166.4	9234.6	10,481**.2**
(b) Small diameter						
Maximum	44.103	43.053	41.32	39.644	39.244	41.4728
Average	28.883	30.112	30.569	28.48	25.946	28.798
Median	35.47	35.122	33.412	31.872	28.919	32.959
Ra	12.993	10.879	8.252	8.731	9.452	10.0614
Rms (Rq)	15.572	13.549	9.908	10.687	11.339	12.211
length	217.96	215.46	215.06	216.87	217.87	216.644
Developed length	253.93	233.13	234.12	235.23	238.09	238.9
Positive Area under curve	6450.9	6569	6620.5	6232.5	5721	6318.78
(c) Folding - classic method						
	φ = ((Developed length a) × (Developed length b)) / ((length a) × (length b))					
	1.26066	1.16922	1.17510	1.16856	1.17303	1.1893

The folding factor for each of the lines is obtained by dividing the corresponding areas from the two tables, then calculating the average of the obtained factors to get a more accurate estimate. Using this calculation method, the folding factor is evaluated about 1.19.

#### Folding factor (second method)

**Fig. 11:**
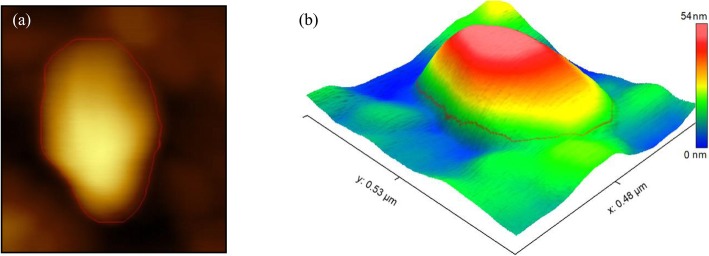
**a** The cross section used to calculate the factor, **b** The 3D view of the cell at the cut-off section

In order to estimate the folding factor, the cell’s perimeter at its cross section was selected and the cell surface area was compared between the smooth and uneven conditions. Figure 11 (a) demonstrates the cross section used to estimate the folding factor and Fig. 11 (b) presents the 3D view of the cell along with the cross section.

As shown in Table 4, dividing these two values results in a folding factor of 1.4, which is greater than the factor calculated by the former method, which was about 1.19. Since the factor obtained by the new technique takes into account the whole surface, it is more accurate than the previous one, which used only the small and large cell diameter in the calculations. The larger folding factor suggests a cell’s surface with more roughness and asymmetry. According to prior studies, unhealthy and cancerous cells are more ruffled than healthy cells. Consequently, this factor can be used as a criterion to identify the cell’s grade of damage.

**Table 4:** The folding factor of the HN-5 cells based on the surface method

Folding - surface method	
Projected area	1873.02 nm^2^
Surface area	2628.13 nm^2^
Folding Factor	1.403

### Viscoelastic properties of the cell

**Fig. 12:**
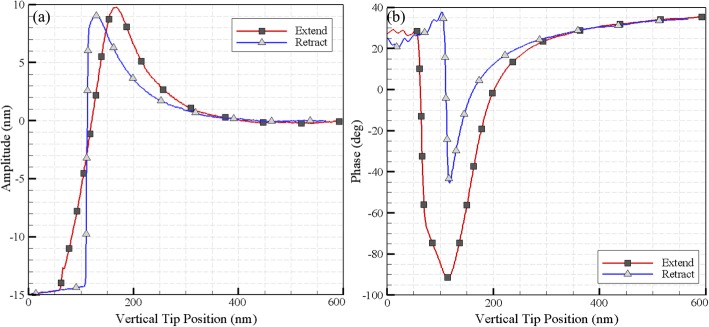
**a** Amplitude and **b** Phase lag extracted for the extend and retract modes of the AFM

In order to obtain the viscoelastic properties of the cell (including spring constant and damping coefficient), the oscillation amplitude, the phase lag far from the sample surface as the oscillation amplitude and the phase lag near the surface, the cantilever frequency, the quality factor far from the sample surface and the calibrated cantilever spring constant are required. Similarly, micro-cantilever data including mean deflection, first harmonic amplitude, and phase lag is needed. In Fig. 12, these measurements for extension and retraction modes of the AFM are presented. Moreover, using information from cantilever’s force-displacement curve, vertical and horizontal displacement, the initial parameters to derive the initial viscoelastic parameters are extracted.

**Fig. 13:**
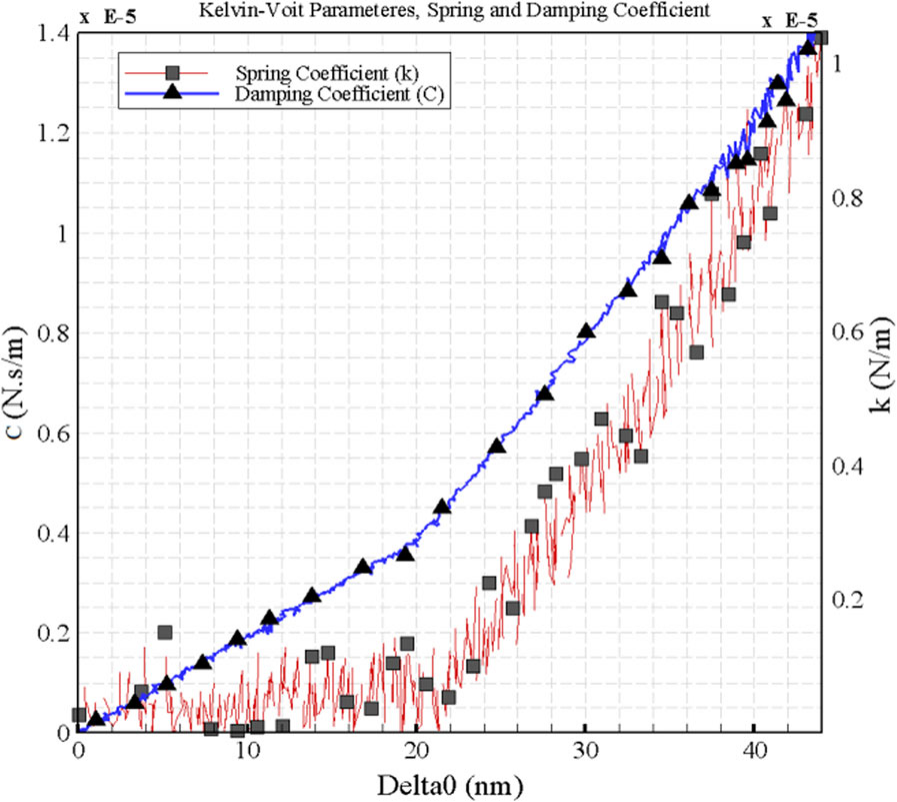
Kelvin-Voigt components (spring constant and damping coefficient) of the HN-5 cell

The indentation depth and the interaction force between the sample and the AFM tip are comprised of two parts; the first is related to quasi-static force and the other is linked to the first harmonic and dynamic force. In order to obtain the value of δ, the oscillation amplitude of the indentation must be summed up with the amount of the tip displacement. Finally, by inserting the parameters in the Kelvin–Voigt equations, the components of the viscoelastic are computed. It is observed that the extracted Kelvin–Voigt parameters depend on the depth of indentation, leading to an increase when the depth increases. The spring constant and damping coefficient of the cell are shown in Fig. 13.

Due to the parallel positioning of the spring and damper in the Kelvin-Voigt model, both components are deformed to an equal extent in response to stress; since the spring prevents extra deformation in the damper. Based on the results, calculated Kelvin-Voigt parameters are reliant on indentation depth; thus an increase in depth leads to the parameters’ growth.

### Creep function

**Fig. 14:**
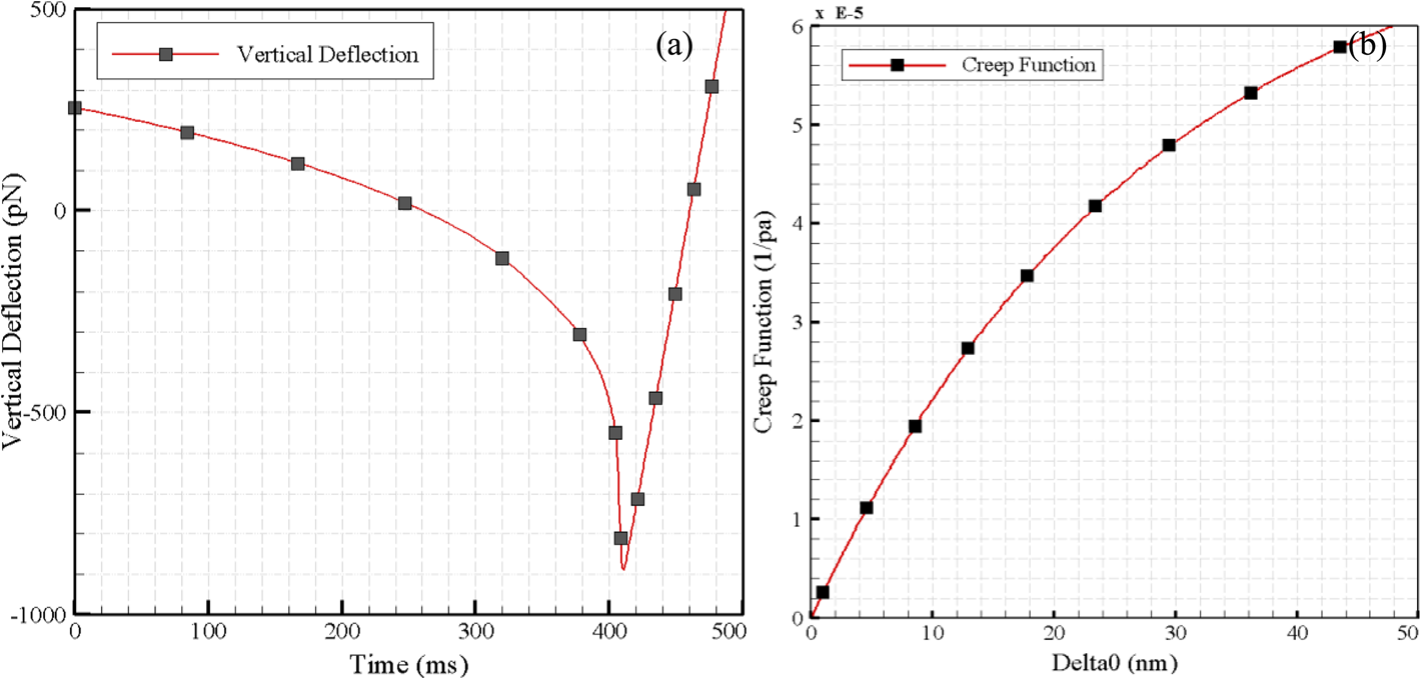
**a** Vertical deflection in terms of the time and **b** Creep function in terms of the oscillation amplitude of indentation

As the cell is simulated using a Kelvin model consisting of a spring and a parallel damper, two other components are required to model the creep function; the modulus of elasticity, with the value of 12 kPa, and time constant of 1.1 s. Finally, the creep function is obtained based on the indentation depth. Figure 14 shows the depth-time graph and the values of the creep function in terms of the oscillation amplitude of the indentation.

### Van der Waals forces

**Fig. 15:**
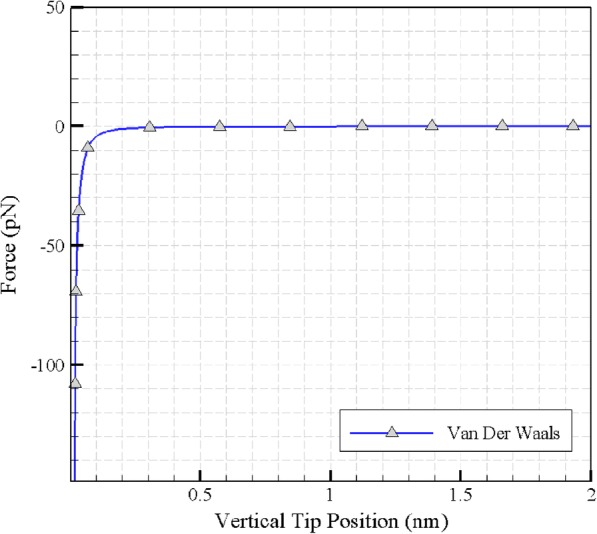
Calculating van der Waals forces using the parameters obtained from the force-depth curve

Calculated Hamaker constants and other required properties are presented in Tables 5, 6 and 7. By extracting these numbers and applying them to the van der Waals formulation, the graph of the force is plotted as presented in Fig. 15.

**Table 5:** The dielectric constant of the medium and Hamaker constant at 25 degrees Celsius [20]

Biological medium	The dielectric constant (*ε*_3_)	Hamaker constant at 25 degrees Celsius (×10^−23^)
Water	80.1	9.02

**Table 6:** Cell and tip characteristics at 25 ° C and pH = 7.3

Material	Modulus of elasticity (*kPa*)	Poisson’s coefficient	The dielectric constant $\left(\frac{N{C}^2}{m^2}\right)$	Surface electrical potential (*mV*)
Cell	8.5	0.5	–	–
AFM tip	170 × 10^6^	0.3	4	16

**Table 7:** Simulation results in both elastic and viscoelastic modes

State	Viscoelastic		Elastic	
	Critical Time (ms)	Critical Force (μN)	Critical Time (ms)	Critical Force (μN)
Sliding force tip-particle	170	0.638	240	0.714
Sliding force substrate-particle	350	0.9814	690	1.596
Rotational force	–	–	510	1.311

### Simulation of the first phase of 3D manipulation for a biological particle with elastic and viscoelastic contact model

In order to simulate the first phase of manipulation, the force and critical time should be computed. The first step is to specify the initial conditions. Since the movement of the base level forms a distortion in the cantilever at any time, force and momentum formed at the cantilever cause a deformation in the particle. The deformation is calculated using the viscoelastic contact mechanics theory for a spherical particle developed in the previous sections. In the next step, the indentation depth is measured and inserted into the kinematics of the problem. The new deformation in the cantilever and the force applied to the particle are calculated and compared with the forces and resistant momentum of the surface.

**Fig. 16:**
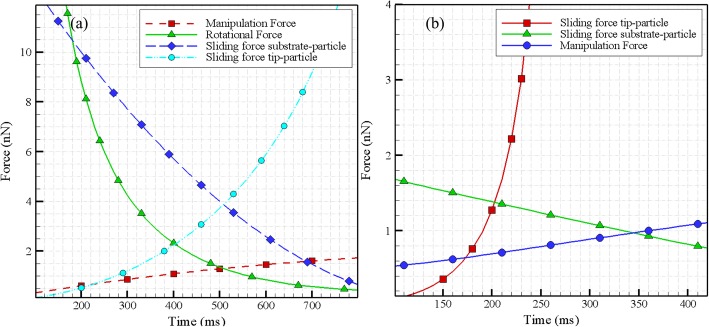
Critical force and time for **a** Elastic and **b** Viscoelastic models for the first phase of 3D manipulation of HN-5

The result of this comparison shows that whether the particle is on the threshold of motion or maintains its adhesion to the surface. Thus, the stated steps are repeated to determine the status of the motion. In Table 7, the simulation result, i.e., critical force and time for elastic and viscoelastic models for the tip sliding on the particle, the particle sliding, and rotation on the substrate are defined. These values are calculated by applying the time constant and the elastic modulus obtained from the experiment. In Figs. 16 (a) and (b), the results are shown for the elastic and viscoelastic states.

In this simulation, the tip and substrate are considered as elastic and the particle as a viscoelastic material. The biological particle is a HN-5 cell, the mechanical properties of which have been presented earlier. Critical force and time are very close to each other in elastic and viscoelastic modes, although these values are slightly lower for the viscoelastic state rather than the elastic state. The decrease in the viscoelastic state causes some of the manipulation force of the particle to be wasted. Although the degree of deformation in the elastic state is greater than the viscoelastic in a similar period of time, the second derivative of indentation in terms of time (indentation acceleration) is less compared to the viscoelastic state and according to kinematic formulas, the amount of force in the elastic state is slightly more than viscoelastic.

### Extractive properties of the extend and retract curves

**Fig. 17:**
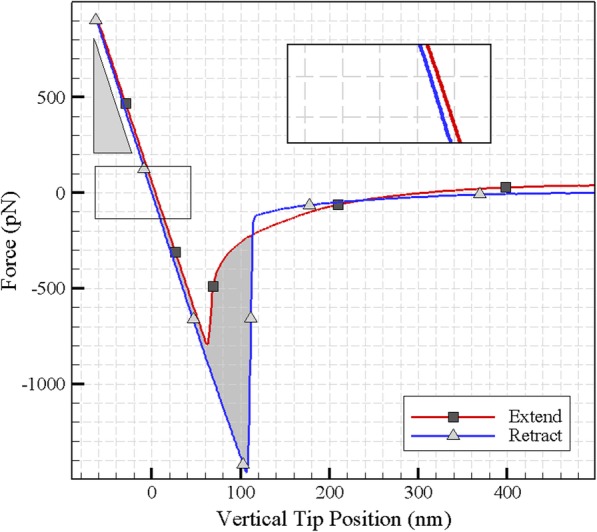
Extractive properties of the extend and retract curves

If the cell does not show hysteresis behavior, there will be no energy dissipation in the retract period of the force-displacement curve, and the extend and retract curves will coincide. However, under the actually experimental conditions they do not coincide and the area between the two curves shows the amount of energy loss. Knowing the amount of energy dissipation helps to obtain a more accurate manipulation method [23, 24]. Another property which can be extracted from the force-depth curve is the stiffness coefficient. Stiffness is the resistance of the sample to the deformation and its dimension is the division of the unit of force by length unit [24, 25]. It can be used as a property for various loading and varied particles and because of specifying the amount of deformation for each particle per applied force, it is an effective parameter in particle manipulation. The deformation resulting from application of force can be elastic or plastic. By applying force and scratching, the tip produces a distortion in the cell. Due to the elasticity of the cell, the larger amount of this deformation is reversible and a small amount of it is permanent and called plastic deformation. In Fig. 17, the coefficient of stiffness, elastic and plastic indentation are observed. Using this parameter, it is possible to predict possible and permanent changes in the shape of a cell due to the forces applied in the manipulation and drug delivery. The extracted values for the above properties are shown in Table 8.

**Table 8:** Energy dissipation, stiffness coefficient and deformation extracted from the extend and retract curve

Plastic Indentation m	Elastic Indentation m	Stiffness coefficient N/m	Energy dissipation J	Cell HN-5
^9−^10 × 3.98	^9−^10 × 6.822	^3−^10 × 14.491	^21−^10 × 47,478	**Amount**

## Conclusion

In this paper, experimental approaches based on AFM were used to extract the physical and mechanical properties of the head and neck cancer (HN-5) cell line. After preparing the equipment and extracting cell geometry and topography, modulus of elasticity was obtained. To calculate Young’s modulus, the indentation was repeatedly performed in at least 10 different points and for the two contact modes. Then, using the Hertz and DMT’s elastic theory, the Young modulus of the cell was calculated at its various points. The results showed that the elasticity modulus is higher in spots with less altitude and vice versa. Likewise, the average of cell’s modulus of elasticity is 12 kPa. The amount of adhesion force was experimentally measured as one of the essential elements in friction force and manipulation development. Similar to the modulus of elasticity, the maximum adhesion was observed on the edges of the cell which are thinner and the minimum adhesion was observed at the center. With increasing the amount of adhesion, more force is needed to detach the cell from the surface, thus increase or decrease in adhesion results in increase or decrease in the manipulation force. For the air medium and cantilever’s contact mode, the average adhesion value was measuered to be 2.47 nN. Moreover, the amount of adhesion work was found to be ~ 98,706 × 10^− 21^ J. In addition, energy dissipation amount in the retract period of the force-displacement curve was ~ 47,478 × 10^− 21^ J.

One more measured characteristic is the folding factor of the cell which was extracted by two diverse methods and found to be 1.19 and 1.40 using them. In order to obtain the viscoelastic properties of the cell, the components of the viscoelastic were computed by inserting required parameters in the Kelvin–Voigt equations. It was concluded that the spring constant and the damping coefficient depend directly on the depth of the indentation. The next obtained characteristic was the creep function. In a viscoelastic material, the creep compliance curve shows that in a constant stress, compliance (or strain) increases over time. The study area in the creep test is related to the part of the delayed elastic strain. It suggests that if the stress is eliminated, the sample will return to its original state over time *t*. Finally, the first phase of 3D manipulation of the cell was simulated. it was found that critical force and time are very close to each other in elastic and viscoelastic modes, although these values are slightly lower for the viscoelastic state compared to the elastic state.

## Data Availability

all relevant raw data on which the conclusions of the paper rely are available.

## Abbreviations

HN-5: The Head and Neck Cancer Cell
AFM: Atomic Force Microscopy
DMT: Derjaguin–Muller–Toporov
JKR: Johnson-Kendall-Roberts
FIEL: Force integration to equal limits
SPIP: Scanning Probe Image Processor
